# Ambident Reactivity in the Phenyl + NO Radical Recombination

**DOI:** 10.1002/chem.202503332

**Published:** 2026-01-30

**Authors:** Virinder Bhagat, Adrián Portela‐González, André K. Eckhardt, J. Philipp Wagner

**Affiliations:** ^1^ Institut Für Organische Chemie Eberhard Karls Universität Tübingen Tübingen Germany; ^2^ Lehrstuhl für Organische Chemie II, Ruhr‐Universität Bochum Bochum Germany; ^3^ Institut Für Organische Und Analytische Chemie Universität Bremen Bremen Germany

**Keywords:** ambident reactivity, matrix isolation, nitric oxide, radicals, two‐state reactivity

## Abstract

The recombination reaction of nitric oxide (NO) with radicals *via* its oxygen atom, rather than the more common nitrogen site, offers an intriguing pathway to the formation of oxynitrenes. The photochemical isomerization of nitroso compounds into their corresponding oxynitrenes may involve such a step. However, because this reaction typically proceeds under continuous irradiation, the formation mechanism has so far remained obscure. In this work, we investigate the recombination of the phenyl radical with NO strictly under thermal conditions, using infrared (IR) and electron paramagnetic resonance (EPR) spectroscopy. A proximal radical pair is generated in a solid argon matrix by photolysis of nitrosobenzene at 254 nm. Upon annealing the matrix to 30 K, the radicals undergo a thermal reaction that yields both nitrosobenzene and phenoxynitrene, notably in comparable amounts. NEVPT2 computations show that phenoxynitrene forms on the 1‐^1^A′ singlet surface, intersecting the 1‐^3^A′′ triplet ground state 1.0 kcal mol^−1^ below the separated reactants. At this crossing point, a substantial spin–orbit coupling (55 cm^−1^) enables two‐state reactivity, thereby illuminating the mechanism behind this elusive transformation.

## Graphical abstract

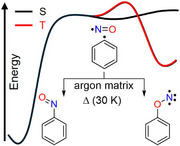

## 1 Introduction

**SCHEME 1:**
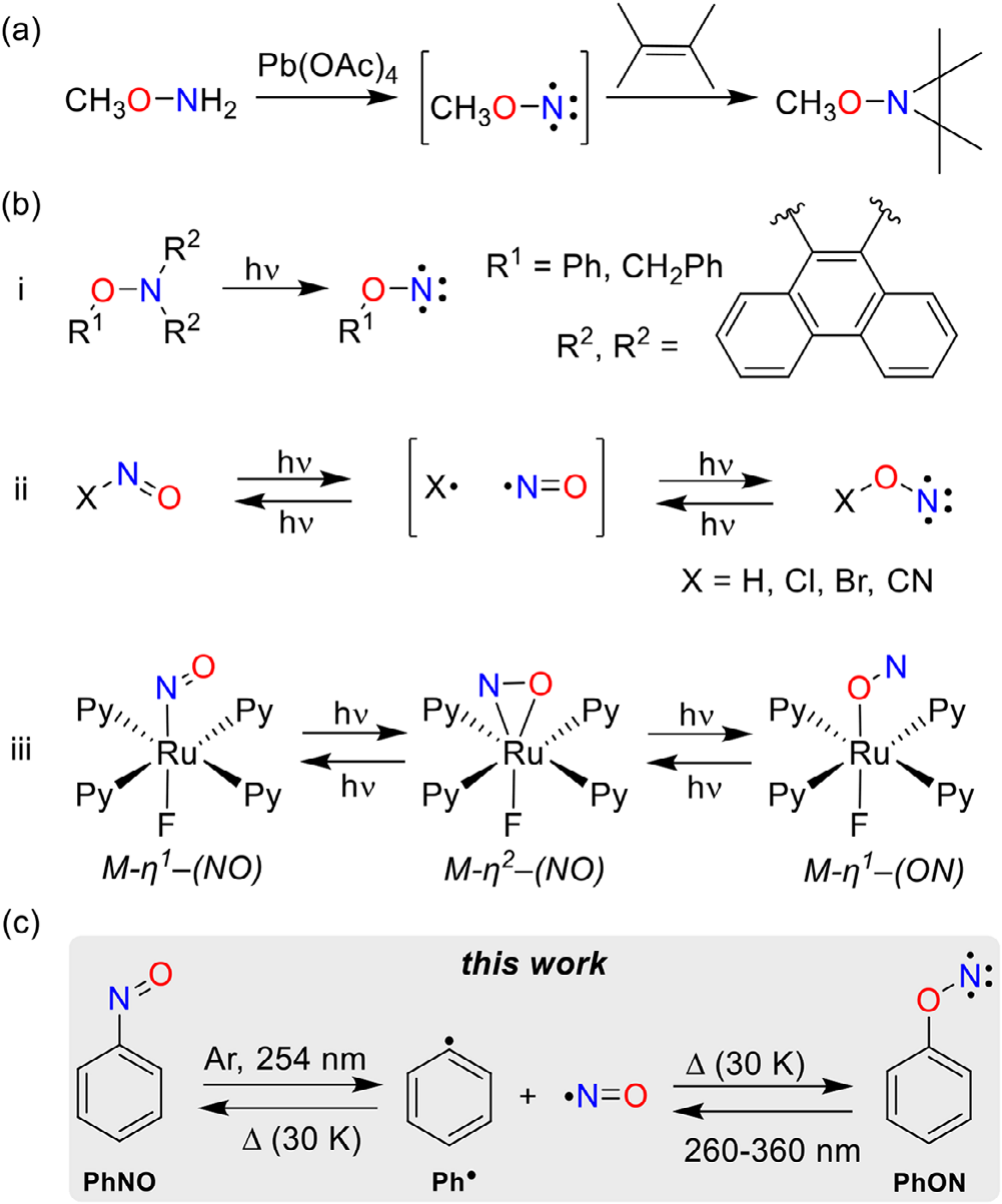
(a) Seminal study trapping an oxynitrene. (b) Photochemical formation of an oxynitrene and the ambident reactivity of the NO radical under photochemical conditions. (c) This work reveals the ambident reactivity of the NO radical in the absence of photochemistry.

Nitric oxide, NO, is a diatomic radical whose reactions predominantly occur at the nitrogen atom [1]. Hartung rationalized this preference recognizing that the interaction of a carbon radical with NO at the oxygen terminus leads to a high‐energy oxynitrene, whereas attack at the nitrogen terminus instead yields a stable nitroso compound [2]. Nevertheless, spin delocalization across both atoms (spin densities: O = 0.286, N = 0.714) [3] in principle allows bonding *via* oxygen, providing a route to otherwise difficult to access oxynitrenes. The intermediacy of such species was first inferred by Brois, who generated methoxynitrene through oxidation of *O*‐methylhydroxylamine with lead tetraacetate and trapped it as an aziridine in 30% yield (Scheme 1a) [4]. Later studies questioned the involvement of oxynitrenes in such reactions due to the nonstereospecific nature of the aziridination reaction [5]; however, this is consistent with the fact that the parent HON is predicted to have a triplet (^3^A′′) ground state [6, 7, 8]. Since then, a number of follow‐up studies have generated oxynitrenes by oxidation of *O*‐substituted hydroxylamines and base‐catalyzed decomposition of *N*‐sulfonyl‐*O*‐alkylhydroxylamines [9, 10, 11, 12].

A different route was followed by Toscano and co‐workers, who described the photochemical formation of oxynitrenes from diazeniumdiolates (R_2_N−N(O)═NOR′), which can either be trapped or rearrange to the corresponding stable oximes [13, 14]. Following this finding, the same group characterized the first organic oxynitrenes, generated in organic glasses from sulfoximine‐ and phenanthrene‐based precursors, and confirmed their triplet ground state by electron paramagnetic resonance (EPR) spectroscopy (Scheme 1b (i)) [15].

Parallel studies explored a complementary route in which nitroso compounds act as photoisomerization precursors to oxynitrenes (i.e., isonitroso compounds) [16, 17, 18, 19]. Under matrix‐isolation conditions, irradiation of the respective nitrosyl isomers produced hydrogen‐, chloro‐, bromo‐, and cyano‐substituted oxynitrenes (HON, ClON, BrON, and NCON; Scheme 1b (ii)). The reversibility of these photoreactions was attributed to transient X···NO (X = H, Cl, Br, and CN) radical pairs [16, 17, 18, 19]. A computational study by Talipov later also pointed out the importance of charge‐transfer excited states in these photoisomerization reactions [20]. Remarkably, ClON and BrON revert to their nitroso forms at temperatures as low as 10 K, likely through quantum mechanical tunneling [16, 17]. The case of the fluoro analog remains unresolved: while Fox et al. initially reported spectral signatures suggesting FON formation [21, 22], later studies found that the assigned spectral bands did not match computational predictions [23] and rather correspond to FNO in different matrix sites [17]. Unfavorable overlap of electronic transitions in FON and FNO was identified as the reason for the inability of photochemistry to provide access to the elusive nitrene [20]. In a related development, the hydroxy‐oxynitrene, HOON, was detected in a supersonic molecular beam [24].

Despite these advances, all known routes to oxynitrenes from nitroso precursors rely on photolysis or other high‐energy activation methods such as electrical discharge. Under such conditions, the possible involvement of NO in the XNO → XON interconversion remains obscured, preventing direct investigation of its ambident reactivity [18, 19]. This dual nitrogen−oxygen bonding character of NO is also central in coordination chemistry, where NO serves as a versatile ligand capable of binding in multiple modes [25, 26, 27, 28, 29]. Such flexibility underpins its utility in applications including data storage [30], holography [31], and photodynamic therapy [32]. For example, ruthenium nitrosyl complexes can undergo wavelength‐dependent interconversion between nitrogen‐bound [M‐η^1^–(NO)] and oxygen‐bound [M‐η^1^–(ON)] isomers *via* an [M‐η^2^–(NO)] intermediate, highlighting a mechanistic contrast to free‐radical photoisomerization (Scheme 1b (iii)) [25].

In the present study, we investigate the ambident reactivity of NO under thermal conditions, focusing specifically on the oxygen‐end recombination. Using nitrosobenzene (**PhNO**) in an argon matrix as a model, we probed whether photochemical excitation is required for oxynitrene formation. While earlier experiments reported only back‐formation of **PhNO** upon annealing of a matrix isolated phenyl···NO radical pair [33], we find that annealing at 30 K enables the formation of the corresponding phenoxynitrene in significant abundance. This process proceeds without photolysis and reflects the high cryogenic reactivity of phenyl radicals (**Ph^●^**) [34, 35], providing direct evidence for the thermal ambident reactivity of NO (Scheme 1c).

## 2 Results and Discussion

**FIGURE 1:**
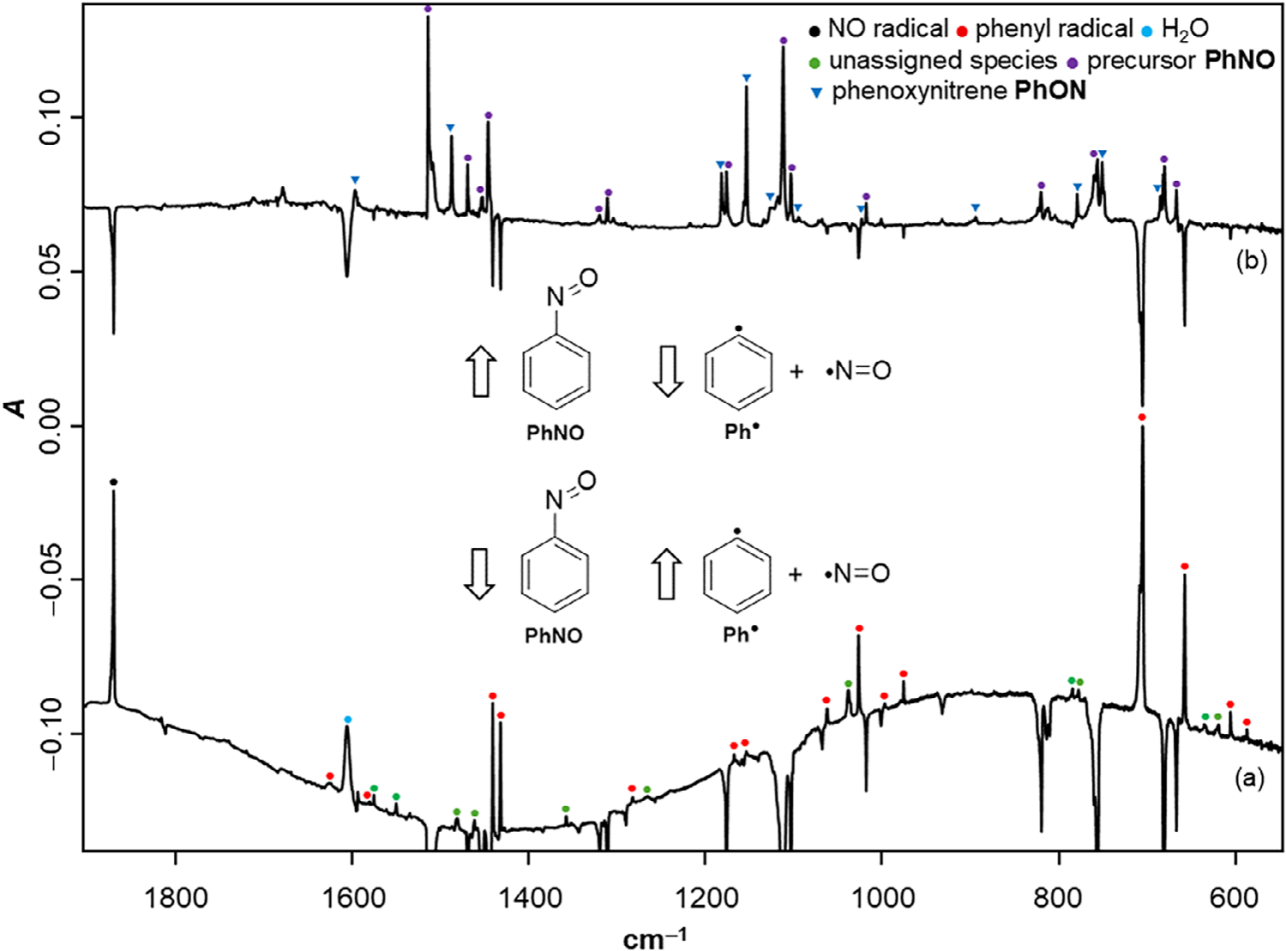
(a) Difference IR spectrum after irradiation of an argon matrix containing nitrosobenzene **PhNO** for 30 min with λ = 254 nm. (b) Difference IR spectrum after annealing the argon matrix containing phenyl radical **Ph^·^** and NO for 15 min at 30 K.

In order to study the ambident reactivity of NO with the phenyl radical **Ph** ^●^, experiments were performed under cryogenic conditions using a matrix isolation setup. The precursor nitrosobenzene **PhNO** was co‐deposited with a large excess of argon onto a cold CsI window at 4.4 K. Afterwards, the deposited matrix was irradiated with *λ* = 254 nm for 30 min, leading to a bleaching of the signals caused by **PhNO** and the appearance of new signals, as shown in the difference spectrum in Figure 1(a). A set of new signals (Figure 1(a), ●) appeared at vibrational energies of 3086, 3070, 3059, 3036, 1626, 1441, 1432, 1283, 1168, 1154, 1062, 1027, 997, 976, 705, 657, 605, and 587 cm^−1^ corresponding to the expected photoproduct phenyl radical **Ph^●^**, and a signal at 1871 cm^−1^ was assigned to the NO radical (Figure 1(a), ●). The IR bands related to **Ph^●^** and NO are in good agreement with previously reported transitions [33]. Alongside the identified bands, a series of low‐intensity features (Figure 1(a), ●) at 1576, 1551, 1481, 1462, 1358, 1266, 1038, 777, and 618 cm^−1^ progressively emerge during irradiation. These bands show no satisfactory correspondence with a known alternative carbenic photoproduct reported in the literature [36] or with plausible isomeric structures thereof (Figure S1); they must therefore remain unassigned, unfortunately.

**FIGURE 2:**
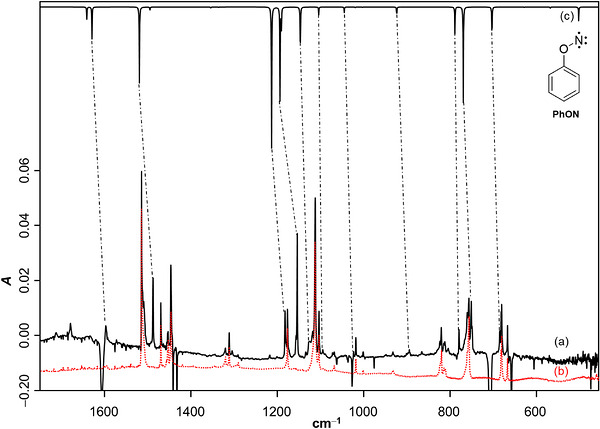
(a) Difference IR spectrum (solid black) after annealing the argon matrix containing phenyl radical **Ph^●^** and NO for 15 min at 30 K. (b) IR spectrum (dotted red, scaled to match the intensities of spectrum (a)) after deposition of nitrosobenzene in the argon matrix. (c) Calculated unscaled harmonic IR spectrum of phenoxynitrene **PhON** at the (U)B3LYP‐D3/def2‐TZVPP level of theory. The vertical lines assign the vibrational transitions of phenoxynitrene **PhON**.

To initiate the thermal reaction between the in situ generated phenyl radical **Ph^●^** and NO, the argon matrix was annealed at 30 K for 15 min. For comparison, we also employed the experimental annealing conditions reported by Kasai et al., who used a higher temperature of 40 K to trigger the same reaction, but observed no discernible difference in the outcome (Figure S4) [33]. We limited our procedures to 30 K, as annealing in an argon matrix above 35 K generally leads to significant matrix evaporation and a reduction in optical transparency [37]. The thermal reaction led to the consumption of both **Ph^●^** and NO, accompanied by the formation of several new species, as depicted in Figure 1(b). One set of emerging IR bands was readily assigned to the precursor compound **PhNO** (Figure 1(b), ●). In contrast, an additional set of IR bands (Figure 1(b), ▼) at 1597, 1488, 1182, 1154, 1126, 1095, 1023, 894, 779, 751, and 685 cm^−1^ was detected that had not been reported in previous experimental observations [33]. Furthermore, this set of IR signals was bleached upon irradiation with *λ* = 260−360 nm for 30 min, resulting in the formation of **Ph^●^** and NO (Figure S2). We surmise that the additional thermal product originates from the reaction between **Ph^●^** and the oxygen atom of NO (**Ph^●^** + ON), producing triplet phenoxynitrene (**PhON**). To corroborate this assignment, the IR spectrum recorded after annealing was compared with the computed spectrum of **PhON** (Figure 2), showing excellent correspondence. Consequently, the additional IR bands were attributed to the formation of **PhON**. Based on the computed intensities of the experimentally observed bands at 1154 and 1112 cm^−1^ corresponding to mixed C─O/N─O stretching vibrations of **PhON** and **PhNO**, respectively, we estimate the product ratio to be 1:2. As summarized in Table S3, analysis of multiple independent vibrational regions yields consistent **PhNO**/**PhON** ratios, demonstrating that the product ratio is not strongly dependent on the specific band pair selected and remains robust despite the typical uncertainties associated with computed IR intensities. Most notably, this ratio qualitatively reflects the relative spin densities at the two reactive centers of NO from which these products originate [3]. This reaction outcome contrasts with the previously reported matrix isolation study, which described only the backformation of **PhNO** [33]. Aside from the use of broadband irradiation to generate the **Ph^●^** ···NO radical pair, the experimental conditions are essentially identical, leaving it unclear why oxynitrene formation upon matrix annealing has not been reported earlier. This difference may arise from the irradiation conditions used in the initial step or from other experimental factors that may have limited the detection of **PhON** in the earlier study.

**FIGURE 3:**
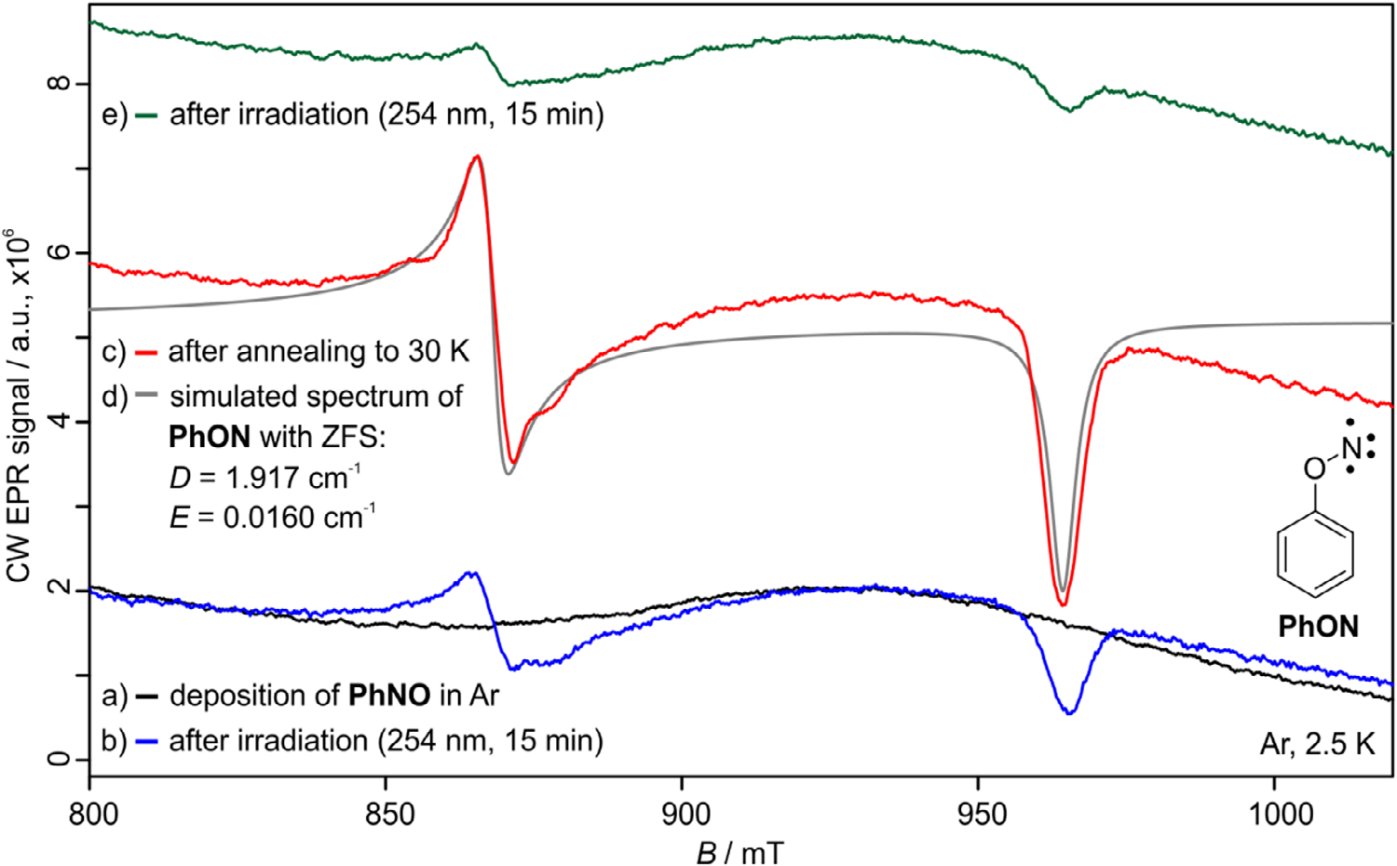
X‐band (ν ≈ 9.8 GHz) EPR spectra (attenuation: 15 dB, modulation amplitude: 20 G, 15 scans) of the photochemistry and subsequent annealing derived from matrix‐isolated nitrosobenzene **PhNO** in Ar at 2.5 K in the 800–1020 mT region. (a) EPR spectrum after deposition of precursor **PhNO**. (b) EPR spectrum after irradiation (254 nm, 15 min) of **PhNO** at 2.5 K. (c) EPR spectrum recorded at 2.5 K after keeping the matrix for 15 min at 30 K after (b). (d) Simulated EPR spectrum of phenoxynitrene **PhON** with ZFS parameters |*D*/*hc*| = 1.917 cm^−1^ and |*E*/*hc*| = 0.0160 cm^−1^, assuming *g* = 2.003. (e) EPR spectrum after irradiation (254 nm, 15 min) of the matrix shown in (c).

The photochemistry of **PhNO** and the subsequent annealing of the resulting species were also investigated with the help of our upgraded matrix isolation EPR spectroscopy setup [38]. This technique offers detailed insight into radical and high‐spin species and is particularly suited for probing the proposed reaction, providing additional confirmation of **PhON** formation as summarized in Figure 3. Irradiation (λ = 254 nm, 15 min) of matrix‐isolated **PhNO** at 2.5 K affords mostly phenyl radical **Ph^●^** [39] (observed in the radical region, Figures S6b, S7b) with additional small signals at 711, 868, and 964 mT (Figure S6b, further details in the Supporting Information). In the radical region, several pairs of bands corresponding to radical pairs between phenyl radical **Ph^●^** and NO were observed (Figure S7a) in agreement with the observations reported by Kasai's group [33].

In analogy to the IR experiment, the matrix was annealed to 30 K and maintained at that temperature for 15 min before being cooled back to 2.5 K, ensuring that the intensities of both spectra are comparable according to Curie's law. After the annealing process, clear changes can be seen in the spectrum (Figures 3c and S5c). The structured spectrum of the isolated phenyl radical **Ph^●^** decreases in intensity (Figure S6c) as well as the complete disappearance of the signals assigned to radical pairs (Figure S7b). Instead, the radical region is dominated by a broad, unstructured band with overall lower intensity (Figure S6c). Concomitantly, the signals initially detected at 868 and 964 mT increase strongly (Figure 3c). These two signals could already be observed after short time irradiation (254 nm, 15 min, Figure 3b) but in a significantly lower intensity compared to the spectrum obtained after annealing (Figure 3c). The two signals exhibiting identical behavior could be simulated using zero‐field splitting (ZFS) parameters |*D*/*hc*| = 1.917 cm^−1^ and |*E*/*hc*| = 0.0160 cm^−1^ (Figure 3d). Therefore, these signals are assigned to phenoxynitrene **PhON** in good agreement with the ZFS parameters previously reported for **PhON** in methylcyclohexane (mCy) glass (*D* = 1.93 cm^−1^ and *E* = 0.0141 cm^−1^) [15].

The ZFS parameters of **PhON** suggest a close proximity between both spins as well as a low rhombic symmetry, which is unusual for nitrenes. Notably, the high *D* value of **PhON** also deviates from an empirical linear correlation presented by Wentrup based on the natural spin density of the nitrene nitrogen atom [40, 41]. This can be understood from a significant spin‐orbit coupling (*SOC*) contribution leading to a higher *D* value. Wentrup argued that the effect might be caused by mixing of the triplet state with the excited zwitterionic singlet state, RO^+^═N^−^ [41].

The detection of phenoxynitrene **PhON** by EPR spectroscopy after annealing the matrix containing phenyl **Ph^●^** and NO radicals confirms their thermal reaction *via* the oxygen end, in agreement with the IR spectroscopic observations. The signals of **PhON** were already detectable after irradiation (Figure 3b), although much lower in intensity, confirming that the generation of **PhON** can also proceed *via* photolysis of nitrosobenzene in analogy to the isomerization reactions of other nitroso compounds [16, 17, 18, 19]. Furthermore, the matrix obtained after annealing to 30 K was exposed to the same irradiation conditions (254 nm, 15 min) used to decompose nitrosobenzene **PhNO** to repeatedly check the photostability of phenoxynitrene **PhON**. Interestingly, the signals assigned to **PhON** clearly decrease upon 254 nm irradiation (Figure 3e) and the signals of phenyl radical are again clearly visible within the radical region (Figure S6d). At the same time, the remainder of the spectrum resembles that obtained directly after the initial photolysis of precursor **PhNO** (Figure S5b,d).

**FIGURE 4:**
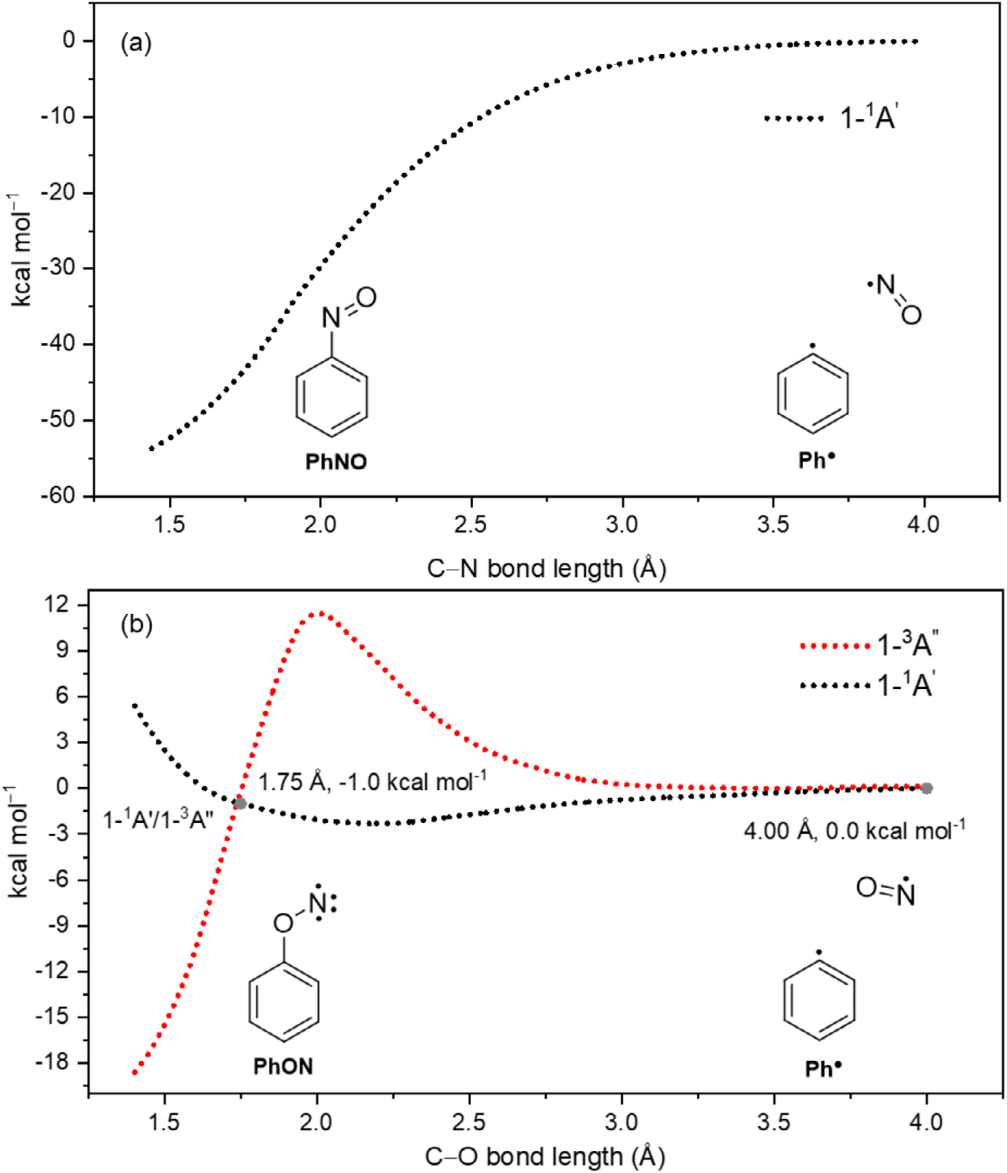
(a) Potential energy scan in the ground 1‐^1^A′ electronic state calculated at the NEVPT2/def2‐TZVPP//CAS(12e,11o)/def2‐TZVPP level of theory with the scan parameter being the distance between the carbon radical center of phenyl radical **Ph^●^** and the nitrogen atom of NO. (b) Potential energy scans (state‐specific) in the 1‐^1^A′ and 1‐^3^A′′ electronic states calculated at the same level of theory with the scan parameter being the distance between the carbon radical center of phenyl radical **Ph^●^** and the oxygen atom of NO.

To gain deeper insight into the experimentally observed C─O bond formation producing phenoxynitrene, we conducted a high‐level computational study. As a first step, we examined the usually observed reactivity of NO through its nitrogen atom (Figure 4a). A relaxed potential energy surface (PES) scan was carried out at the NEVPT2/def2‐TZVPP//CAS(12e,11o)/def2‐TZVPP level of theory for the ground electronic state (^1^A′), using the C–N distance between the carbon atom of **Ph^●^** and the nitrogen atom of NO as the scan coordinate. The resulting PES was found to be entirely downhill from the pre‐reactive complex to the stationary point corresponding to **PhNO**. Therefore, the calculated PES is consistent with the experimental observation of **PhNO** as the main thermal product of the reaction between **Ph^●^** and NO.

Next, we tended to the recombination reaction proceeding *via* the oxygen atom of NO. Thus, we carried out another PES scan using the distance between the carbon atom of **Ph^●^** and the oxygen atom of NO as the scan parameter (C─O bond length); the 1‐^1^A′ and 1‐^3^A′′ electronic states were treated separately in a state‐specific manner (Figure 4b). According to the PES, the formation of **PhON** in the 1‐^3^A′′ state is hindered by a barrier of about 11 kcal mol^−1^, precluding this reaction pathway under the cryogenic conditions applied in our experiments. By contrast, the PES of the 1‐^1^A′ state descends in the direction of the recombination reaction, initially reaching a minimum with a C─O bond distance of 2.20 Å and an energy of −2.3 kcal mol^−1^. Further contraction of the C─O bond length leads only to a minor increase of the energy until the 1‐^1^A′ potential curve reaches a crossing with the 1‐^3^A′′ triplet state at a distance of 1.75 Å at an energy 1.0 kcal mol^−1^ below the separated reactants. The presence of a low‐energy 1‐^1^A′/1‐^3^A′′ crossing point and a high calculated *SOC* of 55 cm^−1^ at this geometry render the population transfer from the 1‐^1^A′ state to the 1‐^3^A′′ a likely event [42, 43]. Beyond the 1‐^1^A′/1‐^3^A′′ crossing, the PES of the 1‐^3^A′′ state decreases monotonically in energy until reaching the minimum corresponding to **PhON**. Hence, the PESs shown in Figure 3b support the feasibility of **PhON** formation under cryogenic conditions *via* a two‐state reactivity mechanism, which offers low‐energy pathways for processes that would otherwise be energetically prohibitive at such low temperatures [44].

## 3 Conclusions

In summary, our study demonstrates that NO exhibits ambident reactivity even at cryogenic temperatures. *In situ* generation of proximal phenyl and NO radicals from nitrosobenzene photolysis, followed by thermal annealing, yields both nitrosobenzene and phenoxynitrene in comparable amounts. These results, supported by high‐level computations, establish thermal and photolytic pathways for phenoxynitrene formation and highlight the dual reactivity of NO. This shows that stable nitroso compounds can be converted into highly reactive nitrenes, pertaining to the ambident nature of NO, which can lead to entirely new reactivity pathways in bimolecular reactions.

## Conflicts of Interest

The authors declare no conflicts of interest.

## Supporting information



Additional Figures and Tables, optimized geometries, and energies.

## Data Availability

The data that support the findings of this study are available in the supplementary material of this article.
