# Supplementary material for: Ambident Reactivity in the Phenyl + NO Radical Recombination
Source: Chemistry. 2026 Jan 30;32(30):e03332. doi: 10.1002/chem.202503332 (PMC13472334; doi:10.1002/chem.202503332)
Supplement: Supplementary file 1 [file CHEM-32-e03332-s001.pdf]

Supporting Information for

Ambident Radical Reactivity: The Phenyl Radical + Nitric Oxide Recombination Reaction

Virinder Bhagat,[a] Adrián Portela-González,[b] André K. Eckhardt,*[b] and J. Philipp Wagner*[a,c]

[a]Institut für Organische Chemie, Eberhard Karls Universität Tübingen, Auf der Morgenstelle 18, 72076 Tübingen

[b]Lehrstuhl für Organische Chemie II, Ruhr-Universität Bochum, Universitätsstraße 150, 44801 Bochum, Germany

[c]Institut für Organische und Analytische Chemie, Universität Bremen, Leobener Straße 7, 28359 Bremen

Email: jpw@uni-bremen.de
Andre.Eckhardt@ruhr-uni-bochum.de

Materials and Methods

Matrix Isolation Experiments

Cryogenic conditions, utilizing a matrix isolation setup, were employed to conduct experiments, which were subsequently analyzed using an infrared spectroscopy setup with a Bruker Vertex 70 spectrophotometer, featuring a resolution of 0.5 cm^{-1}. A SHI CKW-21A closed-cycle helium cryostat was used as part of the matrix isolation setup, which can reach a minimum temperature of 4.4 K. A high vacuum of around 10^{-6} mbar was also maintained using an Edwards oil diffusion pump. The precursor nitrosobenzene (Fluorochem), while being immersed in a cold bath maintained at 0° C, was co-deposited with a large amount of argon (99.9999% pure, Messer-Griesheim) onto a cold CsI window. An MKS mass flow controller was used to maintain a consistent flow rate of the host matrix gases during their deposition. For photochemical transformations, a low-pressure mercury lamp (254 nm, PenRay; pool ray, 11 W) and a high-pressure mercury lamp (USHIO, USH-508S) were used.

The EPR matrix experiments were performed with a ELEXSYS E500 CW-EPR spectrometer with a two-stage RK-415D2 cold head backed by a F-50 Sumitomo cryostat in a setup described in detail in the literature.[1] The X-band EPR spectra were recorded at approximately 9.8 GHz. The field was modulated at 100 kHz and the receiver gain set to 60 dB. The attenuation and modulation amplitude were set to 15 dB (6.45 mW) and 20 G for the full spectra and the nitrene spectra, whereas values of 46 dB (0.0051 mW) and 0.5 G were used to record the radical region (Figure S6) to avoid saturation. The field values were corrected by extrapolation of a reference sample of Mn(II) in ZnS. Nitrosobenzene was obtained from Sigma Aldrich (98%) and used without further purification. The deposition of the precursor was carried out for 30 min with excess argon gas while keeping the sample immersed in a cold bath at 0 °C. Argon (ALPHAGAZ 1 with a purity >99.999 mol% was purchased from Air Liquide).

Computational Details

The B3PLYP functional[2-3] in combination with Grimme’s D3 correction[4] and the Karlsruhe def2-TZVPP basis set[5] was used for geometry optimizations. Vibrational frequency calculations using the harmonic approximation were employed to characterize

the optimized geometries as minima or first-order saddle points, and to obtain zero-point corrections to the electronic energies. Moreover, the calculated IR spectra of different chemical species were also obtained from the vibrational frequency calculations. In addition, the n-electron valence state perturbation (NEVPT2) theory,[6] without the frozen core approximation, was used in combination with the def2-TZVPP basis set for single-point energy calculations at the already calculated stationary points obtained from DFT. All the DFT calculations were carried out using the Gaussian16 quantum chemistry package[7] and for the NEVPT2 calculations, ORCA 5.0.1[8] was employed.

The EPR simulated spectra were fitted from the experimental spectra using the Easyspin[9] package available for Matlab.

Additional Spectroscopy and Computational Data

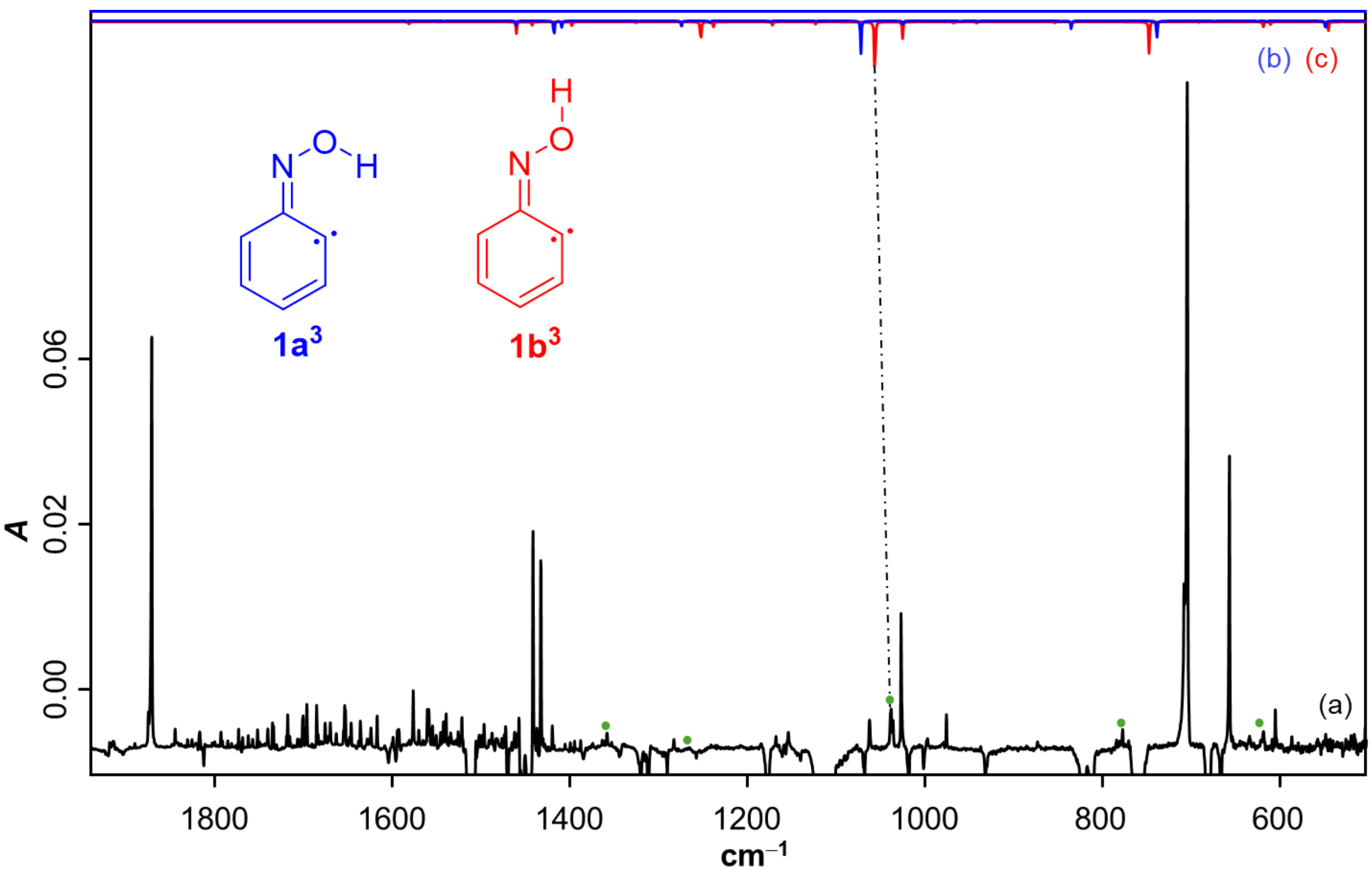

Figure S1. (a) Difference IR spectrum after irradiation of argon matrix containing nitrosobenzene **PhNO** for 30 min with λ = 254 nm. (b) and (c) Calculated unscaled harmonic spectra of triplet **1a** and **1b** at the (U)B3LYP-D3/def2-TZVPP level of theory. Green dots correspond to a previously unassigned photoproduct different from phenyl radical and NO.

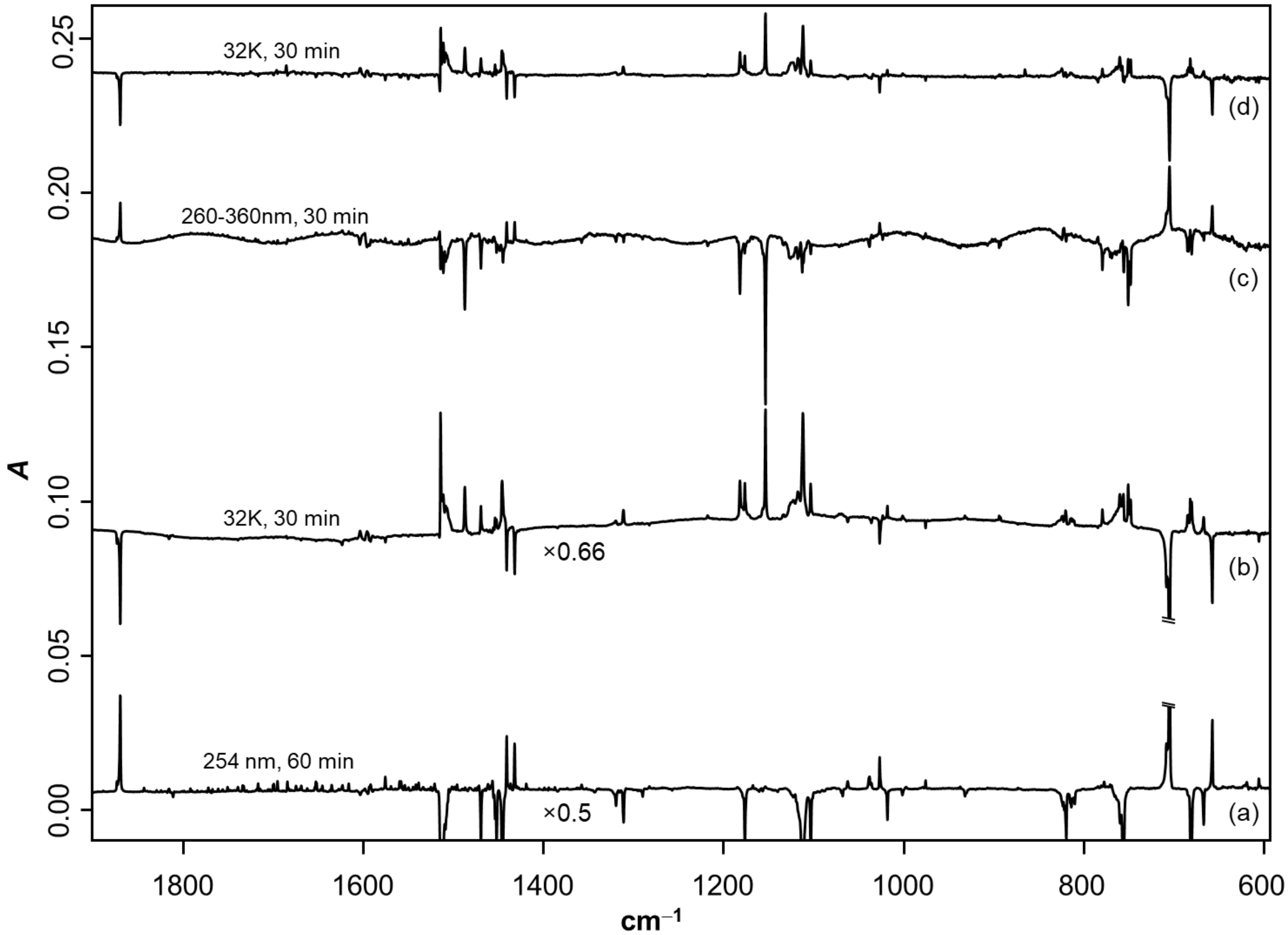

Figure S2. (a) Difference IR spectrum after irradiation of an argon matrix containing nitrosobenzene **PhNO** for 30 min with λ = 254 nm. (b) Difference IR spectrum after annealing the argon matrix from (a) for 30 min at 32 K. (c) Difference IR spectrum after irradiation of an argon matrix containing nitrosobenzene **PhNO** (from experiment (b)) for 30 min with λ = 260-360 nm. (d) Difference IR spectrum after annealing the argon matrix from (c) for 30 min at 32 K.

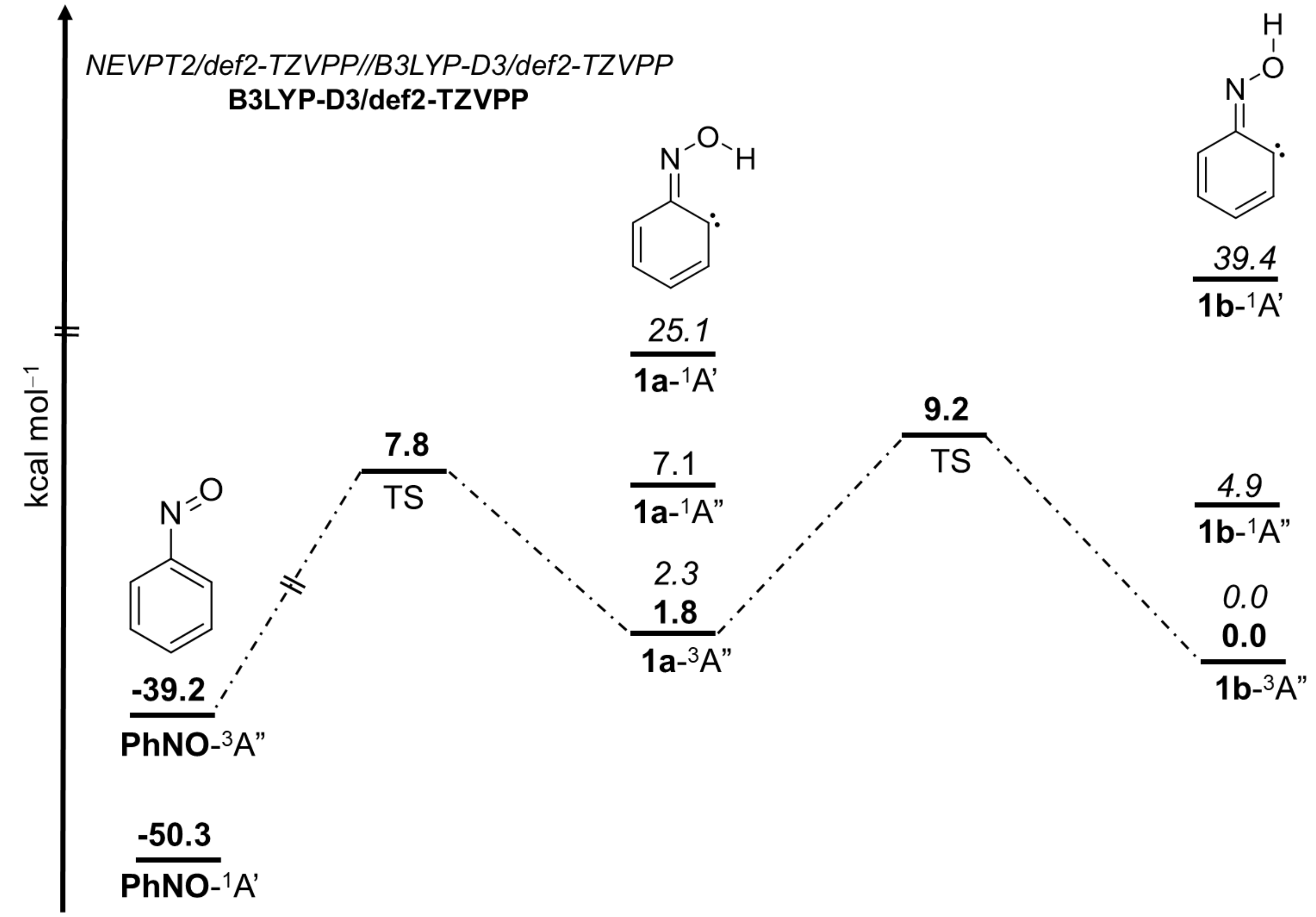

Figure S3. Potential energy surface corresponding to the formation of triplet carbene **1a** and **1b** from the precursor **PhNO**. An active space of 10 electrons and 9 orbitals was used for the NEVPT2 calculations.

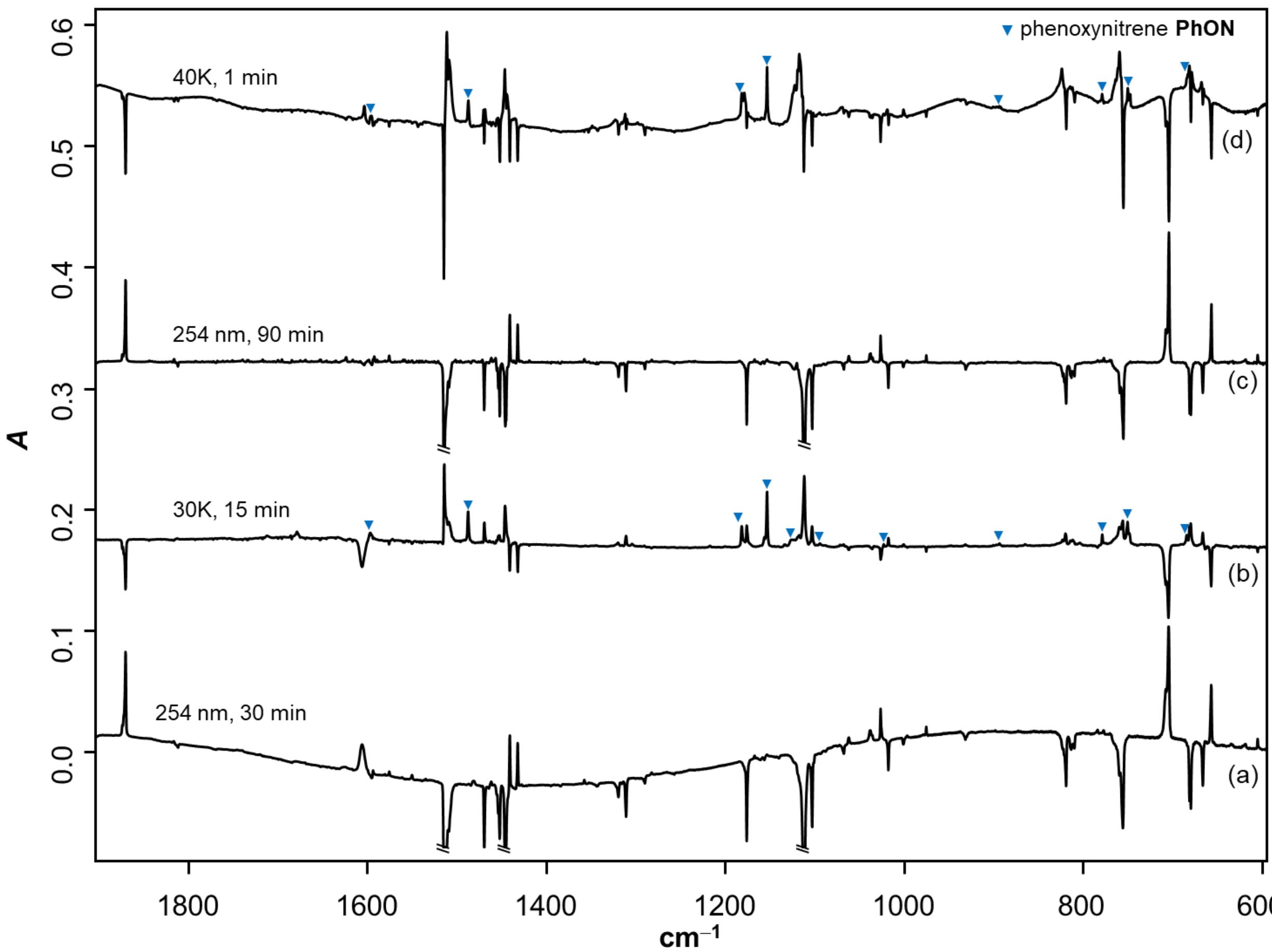

Figure S4. (a) Difference IR spectrum after irradiation of an argon matrix containing nitrosobenzene **PhNO** for 30 min with λ = 254 nm. (b) Difference IR spectrum after annealing the argon matrix from (a) for 30 min at 30 K. (c) Difference IR spectrum after irradiation of an argon matrix containing nitrosobenzene **PhNO** for 90 min with λ = 254 nm. (d) Difference IR spectrum after annealing the argon matrix from (c) for 1 min at 40 K.

IR Band Data

Table S1.
Infrared spectroscopic data of computed (B3PLYP-D3/def2-TZVPP level of theory, unscaled, harmonic) and observed vibrational frequencies of **PhON** in the argon matrix.

Vibrational Mode, ν^a	Experimental		Computed		approx. description
	ν (cm^{-1})	I^b	ν (cm^{-1})	I^c	
33	-	-	3213	0.2	sym. C-H stretch
32	-	-	3205	2.7	sym. C-H stretch
31	-	-	3196	11.4	asym. C-H stretch
30	-	-	3184	8.5	asym. C-H stretch
29	-	-	3175	0.1	asym. C-H stretch
28	-	-	1642	9.3	C-C stretch
27	1597	0.03	1630	19.7	C-C stretch
26	1488	0.041	1520	51.2	i.p. C-H bend
25	-	-	1496	2.3	i.p. C-H bend
24	-	-	1355	0.8	i.p. C-H bend
23	-	-	1336	0.2	C-C stretch, i.p. C-H bend
22	1182	0.03	1214[d]	100.0	i.p. C-H bend, O-N stretch
21	1154	0.085	1194	57.7	i.p. C-H bend, O-N stretch
20	-	-	1191	19.2	i.p. C-H bend
19	1126	0.012	1147	34.9	i.p. C-H bend, C-O stretch
18	1095	0.004	1105	5.0	i.p. C-H bend
17	1023	0.002	1045	3.5	i.p. C-H bend, C-C stretch
16	-	-	1020	0.4	C-C-C trigonal bend
15	-	-	1003	0.2	o.o.p. C-H bend
14	-	-	985	0.0	o.o.p. C-H bend
13	894	0.011	924	5.9	o.o.p. C-H bend
12	-	-	841	0.0	o.o.p. C-H bend
11	779	0.015	789	16.9	i.p. ring deformation
10	751	0.043	770	59.2	o.o.p. C-H bend
9	685	0.011	703	22.0	C-N-C, C-C-C puckering
8	-	-	629	0.2	i.p. ring deformation
7	-	-	568	0.9	i.p. ring deformation, C-O-N bend

6	-	-	503	8.5	C-N-C, C-C-C o.o.p. bend
5	-	-	445	3.6	C-C-O bend
4	-	-	422	0.0	o.o.p. C-H bend

[a]Numbering of the vibrational modes is taken from the Gaussian output; [b]obtained after annealing the argon matrix containing **3** and NO for 15 min; [c]intensity relative to the strongest band; [d]computed absolute intensity: 106.7 km mol^{-1}.

Table S2.
Infrared spectroscopic data of computed (B3PLYP-D3/def2-TZVPP level of theory, unscaled, harmonic) and observed vibrational frequencies of **Ph•** in the argon matrix.

Vibrational Mode, v[a]	Experimental		Computed		approx. description
	v (cm^{-1})	I[b]	v (cm^{-1})	I[c]	
27	3086	0.001	3191	13.5	sym. C-H stretch
26	3070	0.043	3180	28	asym. C-H stretch
25	3059	0.006	3177	5.2	asym. C-H stretch
24	3036	0.005	3165	3.8	asym. C-H stretch
23	-	-	3159	1.3	asym. C-H stretch
22	1626	0.011	1629	2.8	C-C stretching
21	-	-	1573	1.1	C-C stretching
20	1441	0.054	1475	11.7	i.p. C-H bend
19	1432	0.04	1467	7.9	i.p. C-H bend
18	-	-	1326	0.2	i.p. C-H bend
17	1283	0.003	1305	0.5	i.p. C-H bend
16	1168	0.003	1186	0.2	i.p. C-H bend
15	1154	0.005	1182	0.1	i.p. C-H bend
14	1062	0.01	1075	5	C-C stretching
13	1027	0.036	1051	11.6	C-C stretching
12	997	0.004	1017	0.4	ring breathing
11	-	-	999	0.2	o.o.p. C-H bend
10	976	0.007	993	1.8	C-C-C trigonal bending
9	-	-	972	0	o.o.p. C-H bend
8	-	-	899	0.7	o.o.p. C-H bend
7	-	-	818	0	o.o.p. C-H bend
6	705	0.361	724	100[c]	o.o.p. C-H bend
5	657	0.083	676	33.6	C-C-C puckering
4	605	0.011	621	2.2	i.p. ring deformation
3	587	0.003	602	0.8	i.p. ring deformation
2	-	-	428	8.8	C-C-C o.o.p. bending
1	-	-	403	0	C-C-C o.o.p. bending

[a]Numbering of the vibrational modes is taken from the Gaussian output; [b]obtained after irradiating the argon matrix containing the precursor **PhNO** for 30 min with 254nm; [c]intensity relative to the strongest band; [d]computed absolute intensity: 71.4 km mol^{-1}.

EPR spectroscopy

Irradiation (254 nm, 15 min) of nitrosobenzene **PhNO** in Ar produces a spectrum with signals recognizable in the radical region (Figure S6, b) as well as at 711, 868 and 964 mT. Additional broad bands centered at 176, 470 and 558 mT can also be observed in the spectrum (Figure S5, b) but their origin is unknown. The signal at 711 mT resembles the spectrum of an arylnitrene (defined as **X**) with *D* and *E* values of 1.025 cm^{-1} and 0.000 cm^{-1}, respectively. The other signals could not be characterized either (assigned to **Y**) and will be discussed later. Additionally, the spectrum of the phenyl radical **Ph$^\bullet$** can be distinguished by the identification of the most intense peaks compared to the literature but there seems to be a broad, unstructured band overlapping with the **Ph$^\bullet$** spectrum, whose identity is also not clear. After annealing of the matrix, this broad band can be more clearly observed but it is unclear whether it changes upon annealing.

Annealing of the matrix also induces a very slight decrease of the intensity of **X** as well as the disappearance of the signals of **Y**. Separate experiments performed with longer irradiation times show changes in this unidentified bands. On one side, longer irradiation times (1 h) show increased concentration of species **Y**, whereas species **X** is not observed and the broad band below the **Ph$^\bullet$** signals appears to be lower after irradiation but becomes visible after the annealing process. Nevertheless, the disappearance of the **Ph$^\bullet$** signals and the appearance of the signals assigned to phenoxynitrene **PhON** is observed in both cases independent of the irradiation time. Overall, the unidentified species are not believed to take part in the generation of **PhON** and instead, are believed to occur through secondary or tertiary photolysis. This is also confirmed by the increase in the intensity of the unidentified species upon the photolysis (254 nm, 15 nm) performed after the annealing process (Figure S5, d).

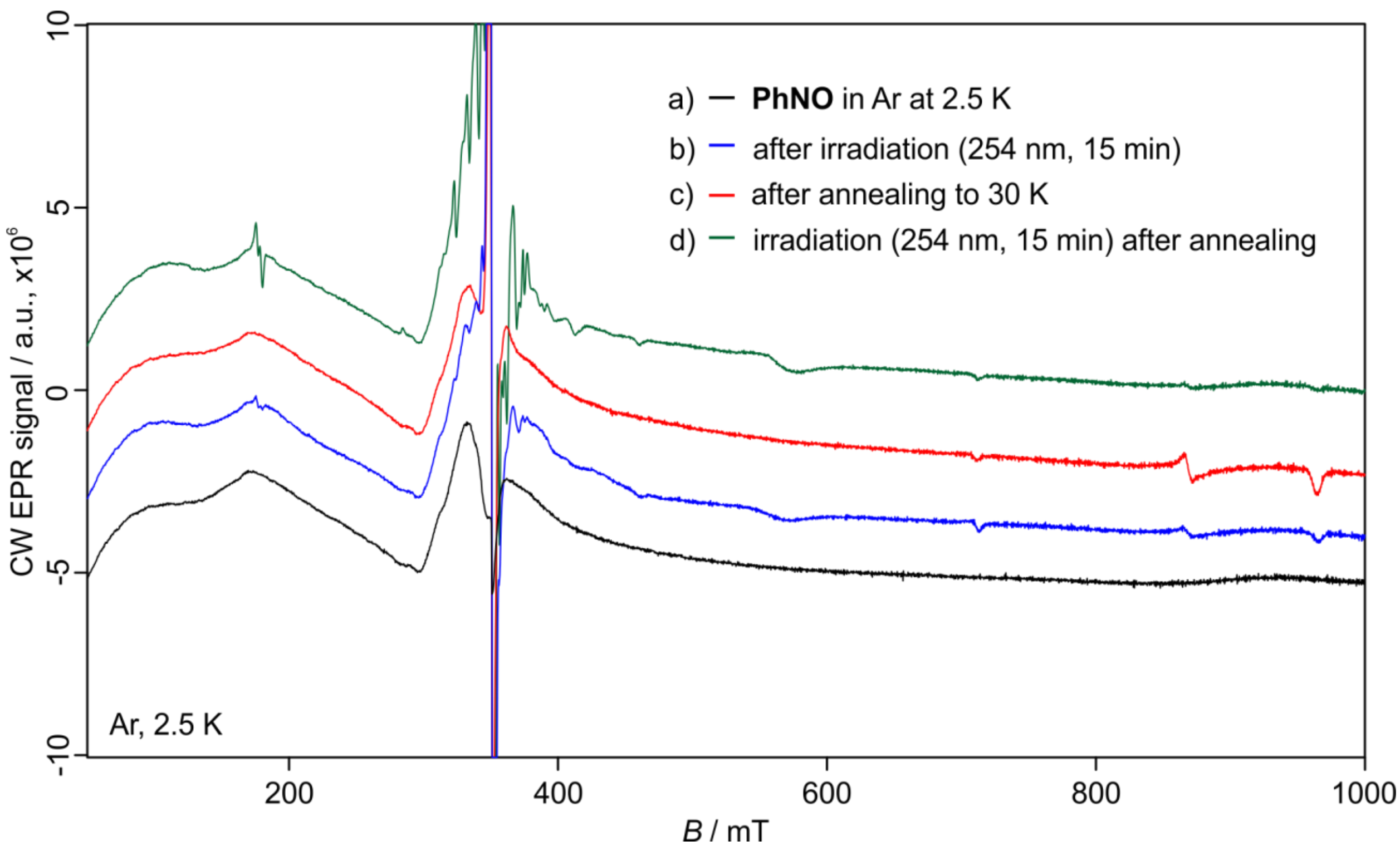

Figure S5. X-band (ν ≈ 9.8 GHz) EPR spectra (attenuation: 15 dB, modulation amplitude: 20 G, 3 scans) of the photochemistry and subsequent annealing derived from matrix-isolated nitrosobenzene **PhNO** in Ar at 2.5 K. a) EPR spectrum after deposition of precursor **PhNO**. b) EPR spectrum after irradiation (254 nm, 15 min) of **PhNO** at 2.5 K. c) EPR spectrum recorded at 2.5 K after keeping the matrix for 15 min at 30 K. d) EPR spectrum after irradiation (254 nm, 15 min) of the matrix after annealing. The bands in the radical region are saturated and are shown in Figure S6 measured with different parameters to avoid saturation.

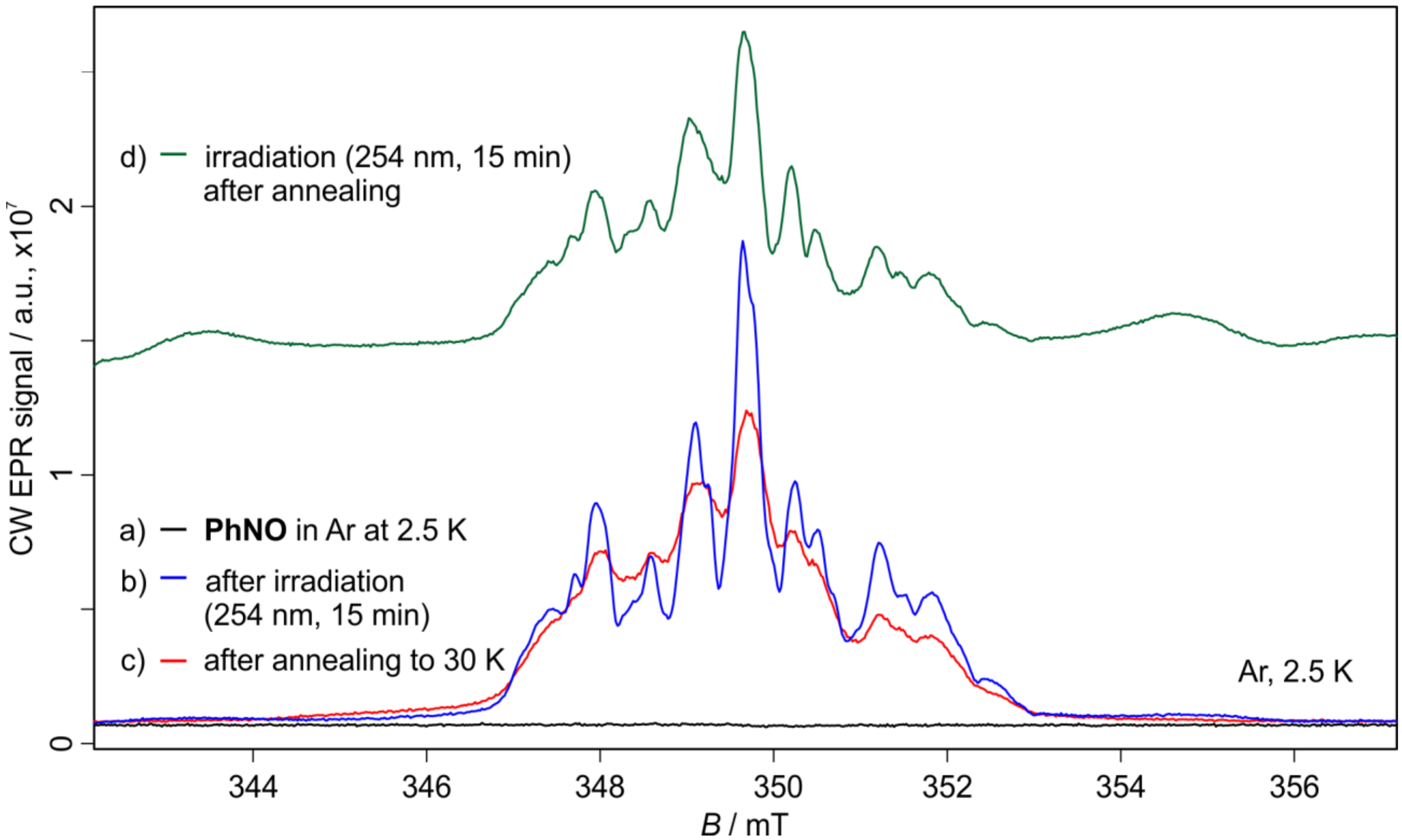

Figure S6. X-band (ν = 9.8 GHz) EPR spectra (attenuation: 46 dB, modulation amplitude: 0.5 G, 10 scans) of the photochemistry and subsequent annealing derived from matrix-isolated nitrosobenzene **PhNO** in Ar at 2.5 K in the radical region. a) EPR spectrum after deposition of precursor **PhNO**. b) EPR spectrum after irradiation (254 nm, 15 min) of **PhNO** at 2.5 K showing the signals of the phenyl radical **Ph$^\bullet$** with an underlying broad structure. c) EPR spectrum recorded at 2.5 K after keeping the matrix for 15 min at 30 K. d) EPR spectrum after irradiation (254 nm, 15 min) of the matrix after annealing showing a more defined structure than after annealing, showing the regeneration of phenyl radical **Ph$^\bullet$**.

Apart from the phenyl radical, around the radical region, the radical pairs of phenyl radical **Ph$^\bullet$** and NO can also be identified by a set of two symmetric signals with respect to the center of the radical region. Three sets of such bands can be recognized at 339.3 and 362.4 mT, 331.4 and 370.6 mT, and 311.7 and 380.9 mT leading to radical pairs with *D* values of 115.5, 196.0 and 418.5 G, respectively. Another potential radical pair at 322.9 and 380.9 mT can be observed leading to a *D* value of 290.0 G, although the higher field band cannot be clearly distinguished. Kasai *et al.* reported radical pairs with separations of 115 and 350 G in their EPR experiments.[10] The radical pair at closer proximity is well

reproduced within our experiment. The differences in the other radical pairs may be due to the different irradiation wavelength and power, as well as matrix preparation.

The assignment of the radical pairs in our experiment is also supported by the disappearance of these signals after irradiation due to the recombination of the pair to form either **PhNO** or **PhON**.

Kasai *et al.* have reported a 9:1 concentration of radical pairs to isolated phenyl radical in their experiments.[10] However, we were not able to provide such information given that the saturation conditions of **•Ph** and the **Ph•**···NO radical pair are different. Therefore, it is not possible for us to quantitatively compare the relative concentration of the free phenyl radical and radical pairs.

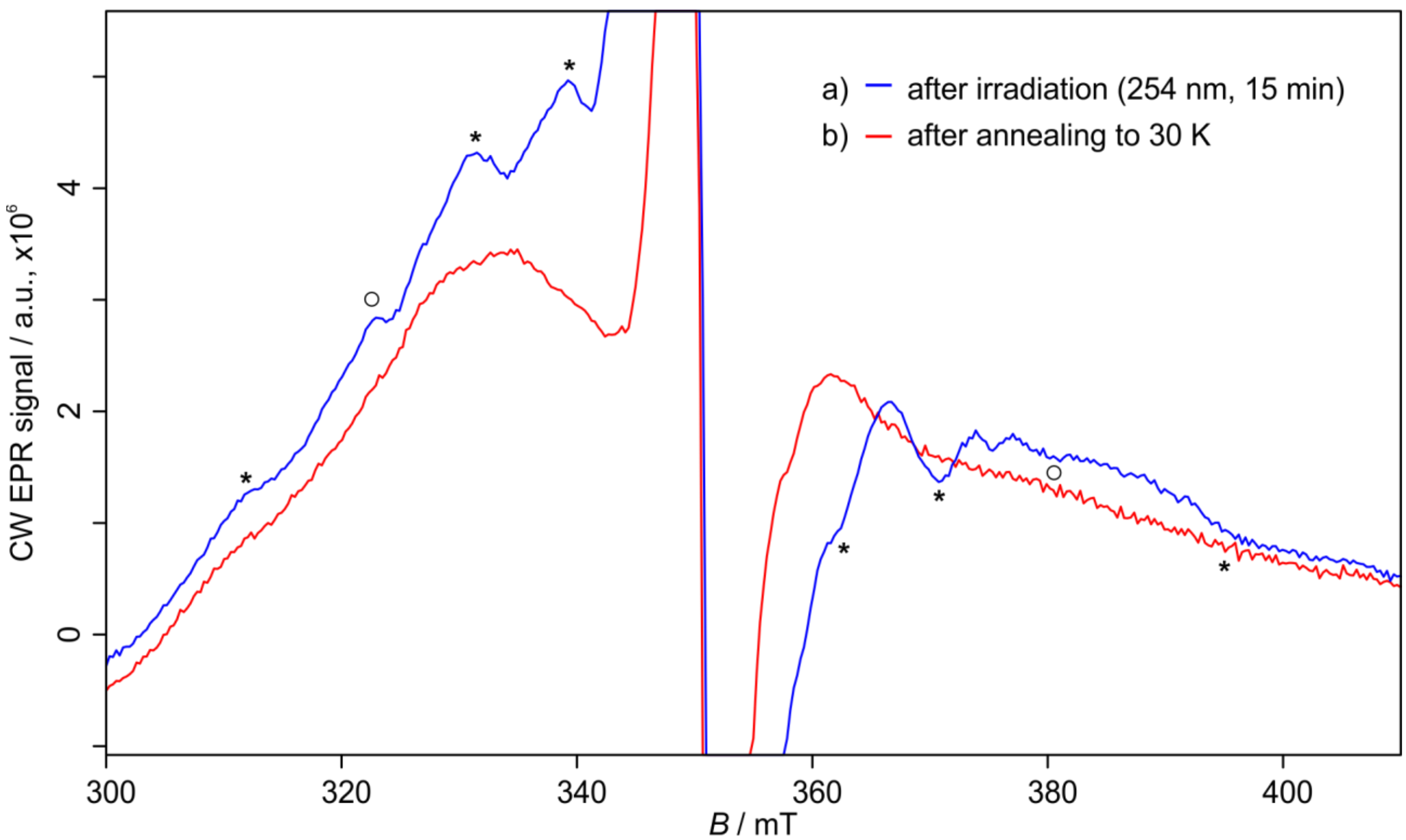

Figure S7. X-band (ν = 9.8 GHz) EPR spectra (attenuation: 15 dB, modulation amplitude: 20 G, 3 scans) showing the **Ph**···NO radical pairs after the photochemistry and subsequent annealing derived from matrix-isolated nitrosobenzene **PhNO** in Ar at 2.5 K in the radical region. EPR spectra recorded after irradiation (254 nm, 15 min) of **PhNO** at 2.5 K (blue trace) and after annealing to 30 K for 15 min recorded at 2.5 K (red trace). The asterisks mark the signal pairs assigned to the **Ph•**···NO radical pairs whereas the circles mark another potential assignment.

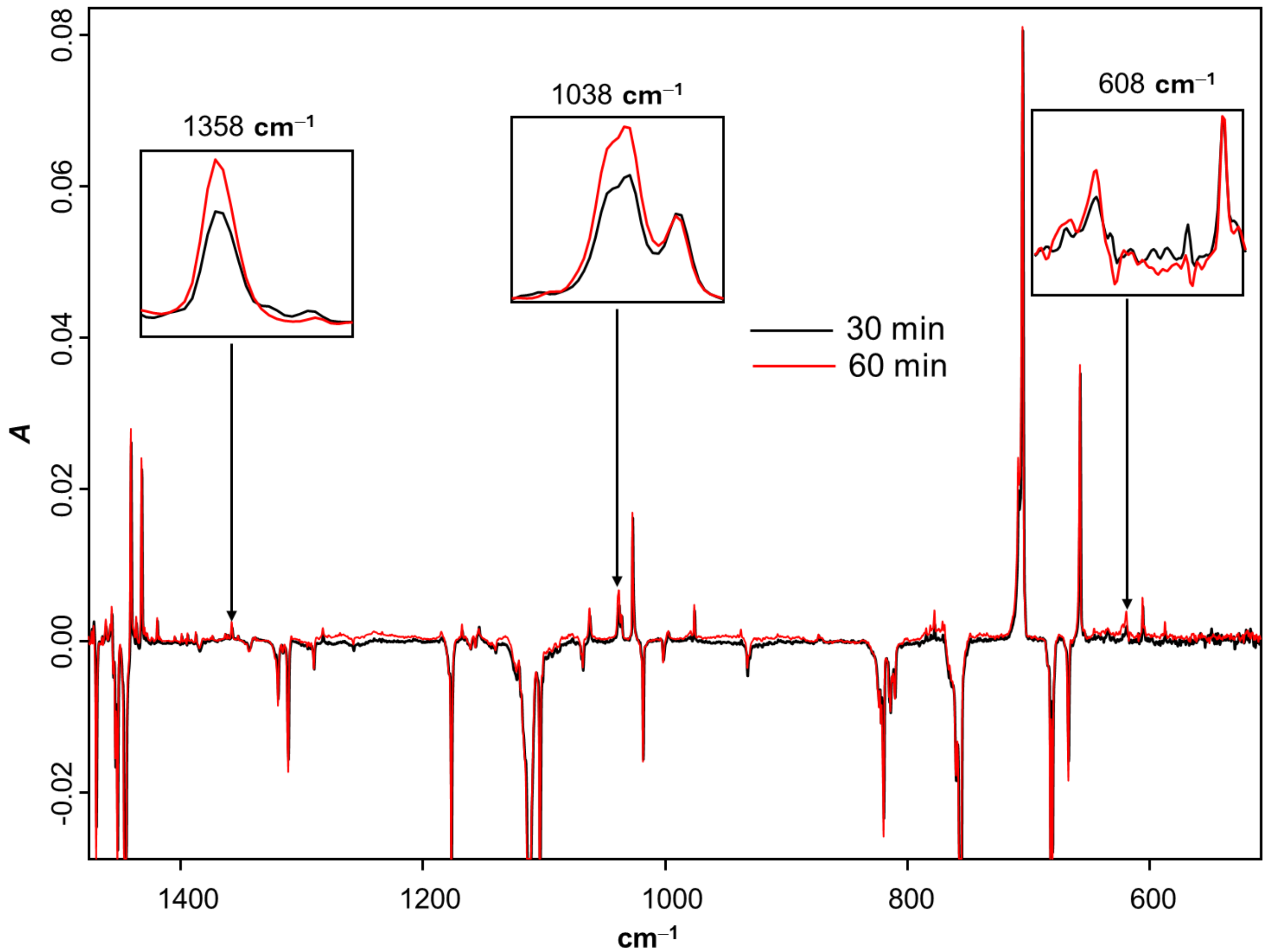

Figure S8. Difference IR spectra after irradiation of an argon matrix containing nitrosobenzene **PhNO** for different intervals of time in two separate experiments with λ = 254 nm. The deposition was done for the same amount of time in both the experiments (see caption Figure 1). The insets correspond to the zoomed view of intense IR bands of the unassigned species. The signals of phenyl radical **Ph•** and NO are adjusted so that they overlay perfectly.

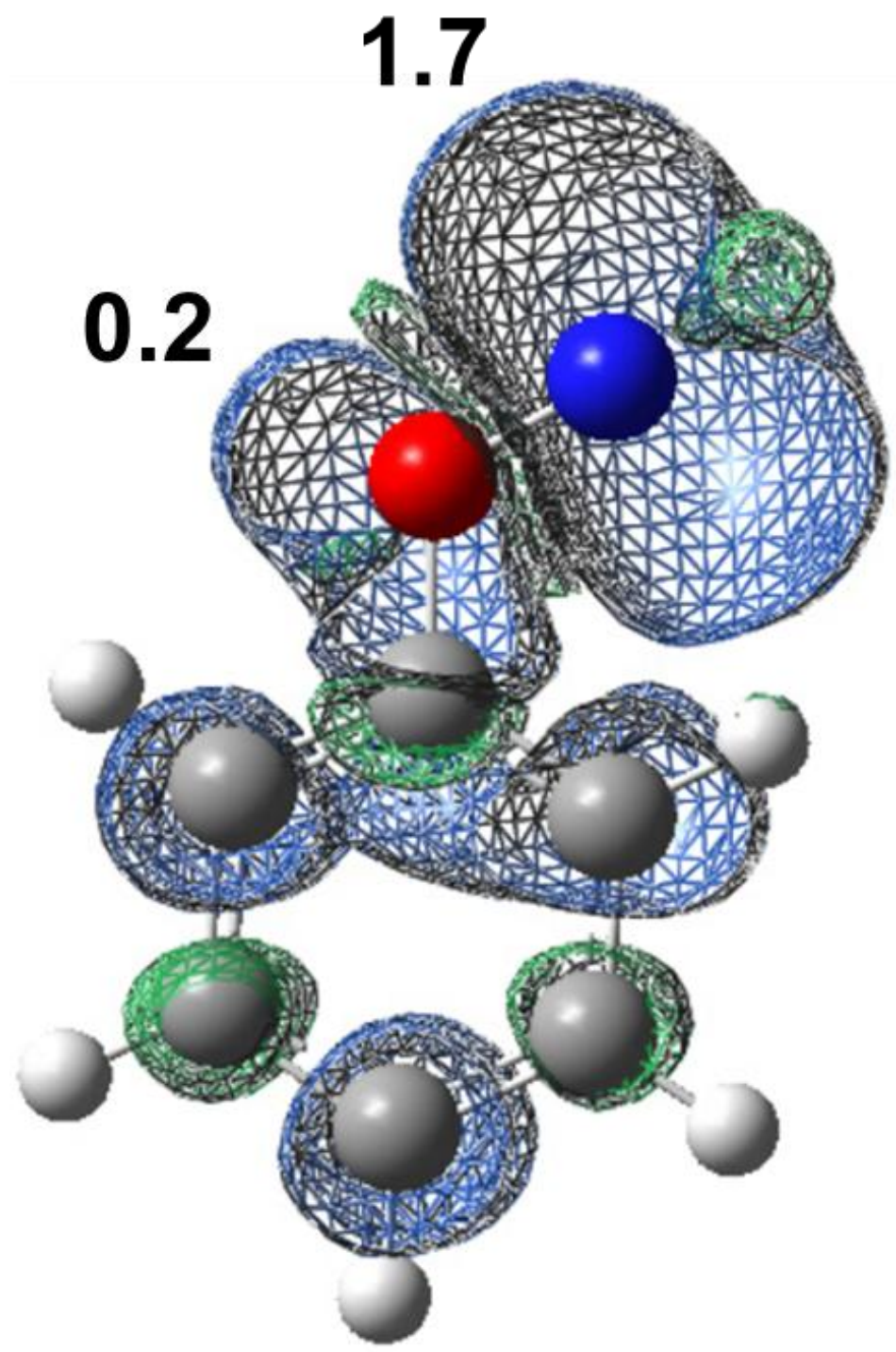

Figure S9. Calculated spin density of triplet phenoxynitrene **PhON**, at different atoms, calculated at (U)B3LYP-D3/def2-TZVPP level of theory.

Table S3.

Estimation of the product ratio (**PhNO:PhON**) resulting from annealing the argon matrix containing phenyl radical **Ph•** and nitric oxide for 15 min at 30 K.

IR band type	band position cm^{-1}(**PhON**, **PhNO**)	Experimental Product Ratio (**PhON** / **PhNO**)
i.p. C−H bend	1488, 1515	1 : 2.22
N−O/C−O	1154, 1194	1 : 1.68
o.o.p. C−H bend	751, 756	1 : 2.02

Cartesian Coordinates and Energies

Table S4.

Cartesian coordinates of optimized geometries calculated at the B3LYP-D3/def2-TZVPP level of theory. Energies are given in Hartree units (E_h) at B3LYP-D3/def2-TZVPP (with ZPVE).

**PhNO**			
-361.592721			
**C**	-2.1681180	0.2578490	0.0000000
**C**	-1.7020810	-1.0525910	0.0000000
**C**	-0.3344420	-1.2940250	0.0000000
**C**	0.5522480	-0.2202530	0.0000000
**C**	0.0899500	1.0981810	0.0000000
**C**	-1.2734130	1.3314050	0.0000000
**H**	-3.2331580	0.4493180	0.0000000
**H**	-2.4007650	-1.8779860	0.0000000
**H**	0.0674550	-2.2985240	0.0000000
**H**	0.8084160	1.9057470	0.0000000
**H**	-1.6502550	2.3455110	0.0000000
**N**	1.9465470	-0.5775440	0.0000000
**O**	2.7247030	0.3494180	0.0000000
**Ph•**			
-231.567819			
**C**	0.0000000	1.3191220	0.0000000
**C**	-1.2095650	0.6300960	0.0000000
**C**	-1.2215530	-0.7696040	0.0000000
**C**	0.0000000	-1.3918250	0.0000000
**C**	1.2215530	-0.7696040	0.0000000
**C**	1.2095650	0.6300970	0.0000000
**H**	0.0000000	2.4009680	0.0000000
**H**	-2.1461810	1.1735230	0.0000000
**H**	-2.1539910	-1.3188540	0.0000000
**H**	2.1539910	-1.3188530	0.0000000
**H**	2.1461810	1.1735230	0.0000000
**PhON**			
-361.540090			
**C**	0.5230680	-2.1162840	0.0000000
**C**	1.5741260	-1.2033010	0.0000000
**C**	1.3185900	0.1610950	0.0000000
**C**	0.0000000	0.5915740	0.0000000
**C**	-1.0669930	-0.2952580	0.0000000

**C**	-0.7896530	-1.6580700	0.0000000
**H**	0.7267980	-3.1781410	0.0000000
**H**	2.5978160	-1.5523790	0.0000000
**H**	2.1165870	0.8898810	0.0000000
**H**	-2.0822240	0.0720250	0.0000000
**H**	-1.6100070	-2.3631440	0.0000000
**N**	-1.3840600	2.4842400	0.0000000
**O**	-0.1769220	1.9829420	0.0000000
**$PhNO^3$**			
-361.575093			
**C**	0.5868810	-2.1242600	0.0000000
**C**	1.6192510	-1.1854200	0.0000000
**C**	1.3385150	0.1686660	0.0000000
**C**	0.0000000	0.5950210	0.0000000
**C**	-1.0478570	-0.3461560	0.0000000
**C**	-0.7398090	-1.6952680	0.0000000
**H**	0.8147320	-3.1811500	0.0000000
**H**	2.6494010	-1.5153490	0.0000000
**H**	2.1282090	0.9067180	0.0000000
**H**	-2.0707430	0.0014070	0.0000000
**H**	-1.5420390	-2.4214440	0.0000000
**N**	-0.2589090	1.9464670	0.0000000
**O**	-1.3386360	2.5136320	0.0000000
**TS_$PhNO^3$_$1a^3$**			
-361.500122			
**C**	0.9300400	-1.9245460	0.0000000
**C**	1.8239470	-0.8511390	0.0000000
**C**	1.3817510	0.4639840	0.0000000
**C**	0.0000000	0.7052390	0.0000000
**C**	-0.8797970	-0.4087910	0.0000000
**C**	-0.4604740	-1.7028100	0.0000000
**H**	1.3040990	-2.9394910	0.0000000
**H**	2.8869800	-1.0506410	0.0000000
**H**	2.0719590	1.2965420	0.0000000
**H**	-1.1510380	-2.5364060	0.0000000
**N**	-0.6328430	1.9060160	0.0000000
**O**	-1.9335980	1.7067060	0.0000000
**H**	-1.9861170	0.5426070	0.0000000
**$1a^3$**			
-361.509623			
**C**	0.6699410	-2.1127690	0.0000000
**C**	1.6694840	-1.1335250	0.0000000

**C**	1.3565370	0.2111450	0.0000000
**C**	0.0000000	0.6366680	0.0000000
**C**	-0.9458180	-0.3974440	0.0000000
**C**	-0.6817510	-1.7323160	0.0000000
**H**	0.9304920	-3.1626320	0.0000000
**H**	2.7081220	-1.4353560	0.0000000
**H**	2.1238840	0.9740950	0.0000000
**H**	-1.4678730	-2.4765290	0.0000000
**N**	-0.2660070	1.9664790	0.0000000
**O**	-1.5922240	2.2596900	0.0000000
**H**	-2.1051420	1.4269860	0.0000000
**TS_1a^3_1b^3**			
-361.497889			
**C**	2.1885630	-0.3705070	0.0074090
**C**	1.7782580	0.9699900	0.0156980
**C**	0.4418930	1.3047490	0.0055230
**C**	-0.5592070	0.2846490	-0.0123050
**C**	-0.0788540	-1.0360280	-0.0164500
**C**	1.2334520	-1.4003150	-0.0110680
**H**	3.2422490	-0.6168500	0.0134030
**H**	2.5240350	1.7533060	0.0275330
**H**	0.1129490	2.3354820	0.0067430
**H**	1.5410360	-2.4383280	-0.0207200
**N**	-1.8460010	0.6742680	-0.0178690
**O**	-2.7098360	-0.4419680	-0.0829670
**H**	-2.8441990	-0.7329710	0.8290190
**1b^3**			
-361.512562			
**C**	0.6144230	-2.1477350	0.0000000
**C**	1.6365770	-1.1915670	0.0000000
**C**	1.3499040	0.1585400	0.0000000
**C**	0.0000000	0.6099190	0.0000000
**C**	-0.9724780	-0.3957520	0.0000000
**C**	-0.7275270	-1.7366500	0.0000000
**H**	0.8535220	-3.2030500	0.0000000
**H**	2.6686520	-1.5153890	0.0000000
**H**	2.1349910	0.9033360	0.0000000
**H**	-1.5288870	-2.4647390	0.0000000
**N**	-0.2086730	1.9471160	0.0000000
**O**	-1.5595440	2.2118830	0.0000000
**H**	-1.5966140	3.1744430	0.0000000

References

[1] A. Portela-González, W. Sander, A. K. Eckhardt, *Angew. Chem. Int. Ed.*, *n/a*, e18517.

[2] A. D. Becke, *J. Chem. Phys.* **1993**, *98*, 5648-5652.

[3] C. Lee, W. Yang, R. G. Parr, *Phys. Rev. B.* **1988**, *37*, 785-789.

[4] S. Grimme, J. Antony, S. Ehrlich, H. Krieg, *J. Chem. Phys.* **2010**, *132*.

[5] F. Weigend, R. Ahlrichs, *Phys. Chem. Chem. Phys.* **2005**, *7*, 3297-3305.

[6] C. Angeli, R. Cimiraglia, J.-P. Malrieu, *J. Chem. Phys.* **2002**, *117*, 9138-9153.

[7] M. J. Frisch, G. W. Trucks, H. B. Schlegel, G. E. Scuseria, M. A. Robb, J. R. Cheeseman, G. Scalmani, V. Barone, G. A. Petersson, H. Nakatsuji, X. Li, M. Caricato, A. V. Marenich, J. Bloino, B. G. Janesko, R. Gomperts, B. Mennucci, H. P. Hratchian, J. V. Ortiz, A. F. Izmaylov, J. L. Sonnenberg, Williams, F. Ding, F. Lipparini, F. Egidi, J. Goings, B. Peng, A. Petrone, T. Henderson, D. Ranasinghe, V. G. Zakrzewski, J. Gao, N. Rega, G. Zheng, W. Liang, M. Hada, M. Ehara, K. Toyota, R. Fukuda, J. Hasegawa, M. Ishida, T. Nakajima, Y. Honda, O. Kitao, H. Nakai, T. Vreven, K. Throssell, J. A. Montgomery Jr., J. E. Peralta, F. Ogliaro, M. J. Bearpark, J. J. Heyd, E. N. Brothers, K. N. Kudin, V. N. Staroverov, T. A. Keith, R. Kobayashi, J. Normand, K. Raghavachari, A. P. Rendell, J. C. Burant, S. S. Iyengar, J. Tomasi, M. Cossi, J. M. Millam, M. Klene, C. Adamo, R. Cammi, J. W. Ochterski, R. L. Martin, K. Morokuma, O. Farkas, J. B. Foresman, D. J. Fox, Wallingford, CT, **2016**.

[8] F. Neese, *Wiley Interdiscip. Rev.-Comput. Mol. Sci.* **2022**, *12*, e1606.

[9] S. Stoll, A. Schweiger, *J. Magn. Reson.* **2006**, *178*, 42-55.

[10] W. G. Hatton, N. P. Hacker, P. H. Kasai, *J. Chem. Soc., Chem. Commun.* **1990**, 227-229.
